# Slowing down DNA translocation through solid-state nanopores by edge-field leakage

**DOI:** 10.1038/s41467-020-20409-4

**Published:** 2021-01-08

**Authors:** Ceming Wang, Sebastian Sensale, Zehao Pan, Satyajyoti Senapati, Hsueh-Chia Chang

**Affiliations:** 1 Department of Chemical and Biomolecular Engineering, University of Notre Dame, Notre Dame, IN USA; 2 Department of Aerospace and Mechanical Engineering, University of Notre Dame, Notre Dame, IN USA

**Keywords:** Nanopores, Fluid dynamics

## Abstract

Solid-state nanopores allow high-throughput single-molecule detection but identifying and even registering all translocating small molecules remain key challenges due to their high translocation speeds. We show here the same electric field that drives the molecules into the pore can be redirected to selectively pin and delay their transport. A thin high-permittivity dielectric coating on bullet-shaped polymer nanopores permits electric field leakage at the pore tip to produce a voltage-dependent surface field on the entry side that can reversibly edge-pin molecules. This mechanism renders molecular entry an activated process with sensitive exponential dependence on the bias voltage and molecular rigidity. This sensitivity allows us to selectively prolong the translocation time of short single-stranded DNA molecules by up to 5 orders of magnitude, to as long as minutes, allowing discrimination against their double-stranded duplexes with 97% confidence.

## Abstract (web summary)

Solid-state nanopores can serve as single molecule sensors for DNA sequencing, but the current designs suffer from fast DNA translocation so low detectivity. Wang et al. slow down and control the translocation speed by 5 orders of magnitude using a leakage field generated at the nanopore tip.

## Introduction

Solid-state and protein nanopores are an emerging class of single-molecule sensors for DNA sequencing^1–3^, protein detection^1,4,5^, and DNA–protein complex analyses^6^. The Achilles heel of nanopores has been the inability to control the motion of biomolecules during voltage-driven translocation through the pore^1,3,7–9^. With the development of enzyme-based methods that ratchet polynucleotides through the pore, the first nanopore-sequencer has been realized using protein nanopores^3^. Despite the progress that has been made with biological nanopores, solid-state nanopores with high stability and tunable pore diameters still offer several advantages. They facilitate integration with compact electronic/optical sensor modalities and allow higher throughput than their protein counterparts. However, developing solid-state nanopore sensors capable of complete characterization of the translocating biomolecules has been challenging^1,7,9^, primarily because of the fast electrophoretic translocation by highly focused electric fields at the pore. The high fields are due to the nanoscale pore dimensions necessary for resistive current signals above thermal noise, and the minimum bias voltage (20–60 mV)^10^ necessary to overcome barriers due to conformation entropy, electrostatic repulsion, and electro-osmotic flow^11,12^.

Typical electrophoretic velocities of nucleic acids across solid-state nanopores are 10–1000 ns per base^1^. At these high velocities, short nucleic acids (<100 nt) as well as small protein molecules are often undetected, much less identified^13^. Thus, a high signal bandwidth (>1 MHz) is needed to fully resolve the resistive pulses^14,15^. High signal bandwidth, however, also strongly amplifies thermal noise in the current recordings; this noise makes the signal resistive pulses become undetectable^16^. This limitation hence prevents accurate profiling of promising cancer biomarkers like proteins, short mRNA fragments, and microRNAs (19–22 nt) by solid-state nanopores^13,17,18^. For the proposed sequencing applications by quantum tunneling, speed control is also a key issue for realizing practical quantum sequencers^9^. An additional mechanism to dramatically reduce (by orders of magnitude) and control the fast electrophoretic velocity of molecules is therefore necessary to realize sensitive and selective solid-state nanopore sensors for short nucleic acids, and other small biomolecules^13^ and sequencing platforms^2,19^.

Multiple approaches have been proposed to slow down the translocation events^20^, which involve either modifying the properties (mostly viscosity) of the electrolyte^10,21,22^, incorporating optical (or magnetic) traps or tweezers^20,23,24^, or using protein tags to slow down the motion of the smaller molecules^25–27^. In the last few years, surface charge density modulation has also been suggested to slow down translocation events^28–33^, mostly by building nanopores with dielectric materials like Al_2_O_3_^29,32,34,35^ and HfO_2_^30^, or by exploring optoelectronic control of surface charge^33^. However, these modifications produce a gating field that is much weaker than the driving field along the pore and are only capable of reducing the translocation speed by at most one order of magnitude^29,30,33,35^, which is small compared to the typical two-decade wide bandwidth of the Poisson distribution of translocation times. Interference with the sensing current signals is also a problem^9^. An intriguing approach has been reported that uses the leakage electric fields to manipulate and preconcentrate DNA in nanofluidic channels^36^. Since the electric field can leak through high-permittivity materials, the leakage field enters the solid surface as an intense normal field that can arrest the transport of biomolecules and trap them at the surface.

In this article we will show that the deposition of a high-permittivity Al_2_O_3_ film over an insulating bullet-shaped polymer nanopore allows the field to leak through the dielectric material and into the upper membrane, producing a field comparable in intensity to the translocation field yet sufficiently weak to prevent permanent trapping of the molecules (and clogging of the nanopores). By properly tuning the bullet-shaped geometry, which has a conical base and a short straight pore at the tip, this field can induce a net voltage-dependent surface charge density on the upper membrane, which can reversibly edge-pin flexible translocating molecules, rendering molecular entry into the pore an activated process. The translocation time becomes a strong function of the molecular rigidity, which is ideally suited for discriminating between short (<100 nt) single-stranded and duplex nucleic acids whose persistence length differ by 2 orders of magnitude. We can selectively prolong the translocation time of short single-stranded DNA molecules by 5 orders of magnitude, thus allowing discrimination against their double-stranded duplexes with 97% confidence. Since the leakage field is outside the nanopore, it does not interfere with the resistive signal current from within the pore tip.

## Results

### Electric field leakage through dielectric materials

Ideal dielectrics are assumed to be perfect insulators^37^ (that is, they present infinite resistivity). However, in reality, their resistivity is finite, leading to a passage of current when subjected to applied voltages commonly known as current leakage^37,38^. This leakage is often undesirable, as it decreases the effective electric field needed for the functioning of multiple devices and promotes material degradation processes^38–40^. In materials often used in nanopores, such as SiO_2_, Si_3_N_4_ and Al_2_O_3_, this leakage is often associated with Poole-Frenkel effects^37,41^ and it manifests at voltages^42^ of the order of 10^9^  V  m^−1^. There is no significant current leakage at lower electric fields. However, due to the finite permittivity of these materials, field lines can penetrate the dielectric film leading to significant field leakage^43,44^.

Optical and electric intensity can become singular at metallic or dielectric cones or wedges. These singular fields are present in tip plasmonics^45^, knife-edge scattering^46^, Taylor cones of electrified drops^47^, etc. In our earlier microchannel electro-osmosis work, the singular tangential electric field at a 90-degree turn of an insulating wall was converted into a comparably singular leakage field across the corner by introducing finite wall permittivity^43,44^. The leakage field exits the other side of the corner as an intense normal field that can arrest the transport of micro-colloids and trap them at the upstream side of the corner. This same mechanism can be incorporated into nanopore devices, leading to high electric fields normal to the upper membrane of the nanopore which are capable to pin the molecules to the tip of the nanopore.

**Fig. 1:**
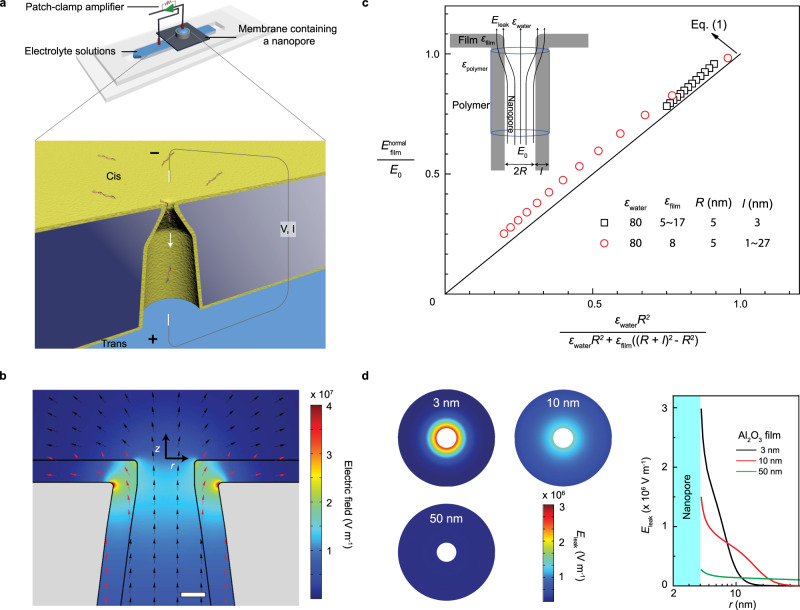
The electric field leakage effect in Al_2_O_3_-coated polymer nanopores. **a** Set-up to measure resistive pulses from the translocation of individual DNA molecules through single bullet-shaped polymer nanopore coated with a thin Al_2_O_3_ layer. **b** Electrostatic modeling of an Al_2_O_3_-coated bullet-like nanopore (tip diameter: 8 nm, half cone angle: 8°) simulated with an applied voltage of 500 mV. Electric field direction and intensity were evaluated numerically on the tip side of the Al_2_O_3_-coated polymer nanopore. The electric field is significantly enhanced and develops a normal field leakage near the sharp pore edge. Scale bar = 3 nm. **c** Validation of Eq. (1) through the use of finite-element-method simulations for normal field leakage in the dielectric film. Different permittivities (black squares, ε_film_: 5~17) and film thicknesses (red circles, *l* : 1~27 nm) were sampled. Inset shows the schematics of a high-permittivity dielectric film on an insulating polymer nanopore orifice and the Gauss volume used to estimate the leakage field around the pore. **d** Left: Surface plots of the strength of normal leakage field (*E*_leak_) showing the normal leakage field at the pore edge is a strong function of Al_2_O_3_ film thickness (nanopore diameter, 8 nm). Right: Axial dependence of the normal leakage field as a function of distance from the pore mouth r for three Al_2_O_3_ film thicknesses.

Our designed solid-state nanopore has a conical base and a short straight pore at the tip, which is coated with a highly conformal Al_2_O_3_ film (see Fig. 1a and Supplementary Fig. 1). The conical base with the insulating PET (polyethylene terephthalate) membrane wall focuses the electric field and the high-permittivity Al_2_O_3_ film on the straight pore edge facilitates field leakage at the tip end (see Fig. 1b). Once the field lines enter the dielectric film inside the pore, the axially conditioned parallel field lines within the straight pore region ensure that the field intensity in the dielectric film is identical to that in the aqueous phase in the pore, despite the higher permittivity of the latter phase. With the converging geometry at the conical base, the field lines are confined to the aqueous bulk. A simple Gauss volume flux balance then allows us to relate the normal leakage field *E*_leak_ and the average electric field *E*_0_ in the pore entrance at the neck with the conic base,

1 $$E_{{\mathrm{leak}}} = E_{{\mathrm{film}}}^{{\mathrm{normal}}}\left( {\frac{{\varepsilon _{{\mathrm{film}}}}}{{\varepsilon _{{\mathrm{water}}}}}} \right) = \frac{{E_0\varepsilon _{{\mathrm{film}}}}}{{\varepsilon _{{\mathrm{water}}} + \varepsilon _{{\mathrm{film}}}(\left( {1 + l/R} \right)^2 \,- \, 1)}},$$

where *ε*_water_ and *ε*_film_ are the permittivity of water and dielectric film, respectively, *R* is the radius of nanopore orifice, and *l* is the thickness of the dielectric film (see Fig. 1c). $E_{{\mathrm{film}}}^{{\mathrm{normal}}}$ is the field inside the dielectric membrane, which can be considered to be equal to the field in the liquid region $E_{{\mathrm{water}}}^{{\mathrm{normal}}}$ (near the tip of the pore) for large pores and small values of *l* (see Supplementary Note 1). Two limits of Eq. (1) are instructive. For *l*/*R* approaching infinity, corresponding to a non-polymeric dielectric membrane whose area is much larger than the pore tip area, *E*_leak_/*E*_0_ scales as (*R*/*l*)^2^ ≪ 1. This indicates conventional solid-state nanopores fabricated in dielectric membranes (such as SiN, SiO_2_, and Al_2_O_3_, etc.) cannot produce significant molecule-pinning field at the pore edge, as the field is distributed over a large surface area that scales as *l*^2^. Indeed, to date, there has been no experimental report of prolonging translocation times in dielectric membrane nanopores by edge-field leakage. In fact, this field penetration across the entire dielectric membrane causes significant dielectric noise in the nanopores^14,48,49^. The other limit of *l*/*R* approaching zero yields that *E*_leak_ *~ E*_0_(*ε*_film_*/ε*_water_). The leakage field would then be proportional to the applied field and of comparable intensity if the permittivity ratio is not too small. Hence, a compound pore, with a nearly insulating polymer membrane and a thin high-permittivity dielectric film, is necessary for a leakage field with intensity and dimension that can delay the molecular translocation time without generating significant noise in the resistive signal. Finite-element-method (FEM) simulations confirm that the electric field intensity within the dielectric film increases considerably and develops a normal field leakage when approaching the pore edge. In Fig. 1d, the intensity of normal leakage field along the *r* axis is shown for different film thicknesses *l* (3–50 nm). The rapidly increasing (singular) intensity of normal field at the pore edge with decreasing *l* confirms enhanced field leakage in PET nanopores coated with a thin Al_2_O_3_ film. Note that the zero-thickness limit is singular, as the field at the pore edge would be purely tangential for a perfectly insulating membrane.

To create our nanopores, we fabricate single nanopores with asymmetric shapes in PET membranes by the track-etching method^50^ and subsequently deposit an Al_2_O_3_ dielectric film on the pore wall by atomic layer deposition (ALD)^51^. The strength of the electric field at the pore tip can be fine-tuned through the choice of cone angles under the same applied voltage. The as-fabricated nanopores with large cone angles have a bullet-like shape while nanopores with small cone angles have a trumpet-like shape^52^ (Supplementary Fig. 1). PET is highly insulating and has been used as supporting substrate to significantly reduce the dielectric noise of nanopores in dielectric membranes^49^. Al_2_O_3_ has a large dielectric constant of 8. ALD offers precise control of nanoscale film thickness.

### Field leakage induced delay of DNA translocation

**Fig. 2:**
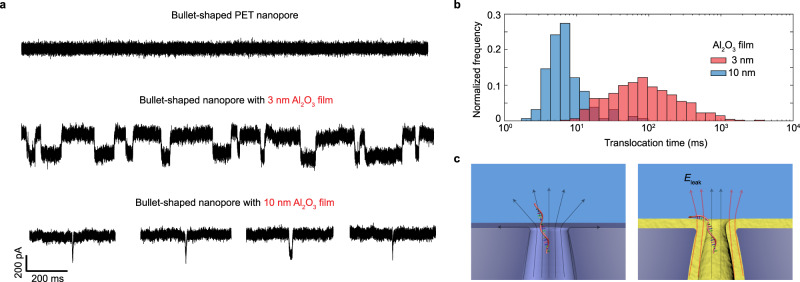
Slowing down ssDNA translocation speed with electric field leakage. **a** Representative current traces for 22 nt ssDNA translocation through a bare bullet-shaped PET nanopore without Al_2_O_3_ film coating (diameter, 16 nm) and two bullet-shaped nanopores coated with 3 nm or 10 nm Al_2_O_3_ film under an applied voltage of 500 mV. Both Al_2_O_3_-coated nanopores have the same final tip diameter (10 nm). All three nanopores have similar bullet-like shapes (half cone angle, 7 ± 2°). Slow translocation of 22 nt ssDNA is observed using nanopores with thin Al_2_O_3_ film coating and the average translocation time is a function of film thickness. **b** Normalized histogram of translocation times for nanopores with 3 nm or 10 nm Al_2_O_3_ film. Average translocation time: 3 nm Al_2_O_3_ film, 159 ms (*n* = 492 events); 10 nm, 13 ms (*n* = 46 events). **c** Schematic showing the dominant tangential electric field at the bare PET nanopore edge results in a fast translocation of 22 nt ssDNA (left) while the normal leakage field at the Al_2_O_3_-coated nanopore edge traps the ssDNA and thus reduces its mobility.

Having fabricated the Al_2_O_3_-coated PET nanopores, we next tested the field leakage effect on the translocation of DNA molecules. We selected 22 nucleotides (nt) long single stranded DNA (ssDNA) molecules as representative small nucleic acid molecules whose fast translocation poses a major challenge to their detection by other solid-state nanopores^17^. Figure 2a presents typical current traces recorded during the translocation of these molecules for a bare bullet-shaped PET nanopore without Al_2_O_3_ film coating and for one with a 3 nm Al_2_O_3_ film coating (diameter 10 nm). Resolvable signals due to translocation events are only observed in bullet-shaped nanopores coated with Al_2_O_3_ films, where substantial field leakage occurs. Moreover, the translocation time strongly depends on the Al_2_O_3_ film thickness. By comparing these two nanopores, we see an increase of one order of magnitude in the observed average translocation time, from 13 to 159 ms (see Fig. 2b), when the thickness of Al_2_O_3_ film decreases from 10 nm to 3 nm. As suggested from our field flux balance, the field leakage at the pore edge is indeed expected to become stronger with thinner Al_2_O_3_ dielectric layer. In contrast, no translocation event is detected for bare bullet PET nanopores without the high-permittivity dielectric layer that sustains field leakage—it is a singular limit. Due to limitations on the detection electronics, translocation of 22 nt ssDNA through the bare bullet PET nanopore is too fast to be detected. We verify this by translocating lambda DNA (48.5 kbp) through the bare pore. We found unique blockage signatures not observed for 22 nt ssDNA. Importantly, the average translocation time for lambda DNA was 2.6 ms, translating into 54 ns per base or 1.2 µs for 22 nt ssDNA, which is undetectable (see Supplementary Fig. 2). The correlation between dielectric film thickness and translocation time is observed in all tested Al_2_O_3_-coated PET nanopores at different bias voltages.

**Fig. 3:**
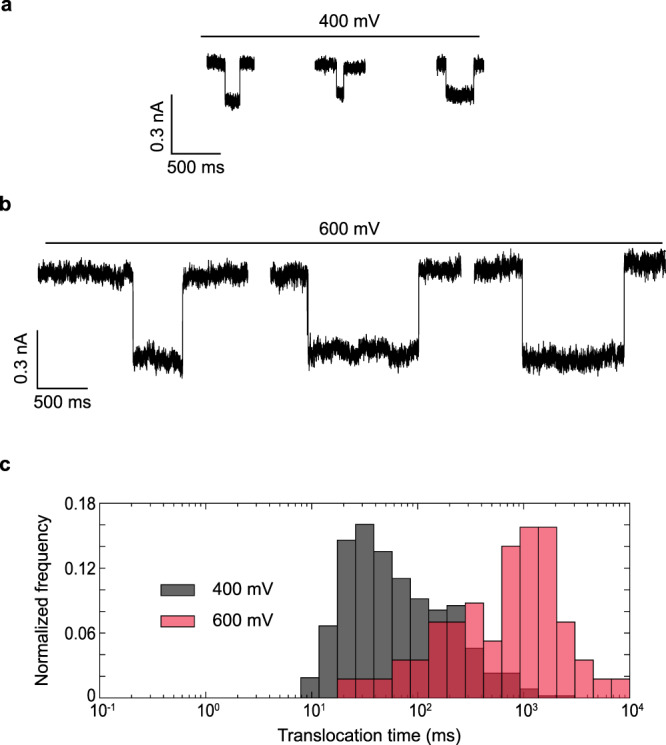
Effects of electric field leakage on ssDNA transport. **a** Representative translocation signals for 22 nt ssDNA translocations at applied voltages of 400 mV. **b** Representative translocation signals for 22 nt ssDNA translocations at applied voltages of 600 mV. **c** Normalized histogram of corresponding translocation times at applied voltages of 400 mV (*n* = 480 events) and 600 mV (*n* = 57 events) and average translocation time as a function of applied voltage. Increasing the strength of electric field leakage can increase the translocation time of ssDNA. Data were acquired using a nanopore coated with 3 nm Al_2_O_3_ film (diameter, 8 nm; half cone angle, 9 ± 2°).

Other than dielectric film thickness, different field leakage strengths can also be fine-tuned by varying cone angles of nanopore or bias voltages, as expected from Eq. (1). Previous studies have shown that the translocation of 100 nt ssDNA can be slowed down to ~0.18 ms using solid-state nanopores in Al_2_O_3_ membranes^29^. We observed an average translocation time ~125 ms for 22 nt ssDNA in Al_2_O_3_-coated PET nanopores (half cone angle ~9 ± 2°, diameter 8 nm, applied voltage 400 mV). Due to confinement effects and electrostatic interactions, translocation times of DNA molecules (and other small molecules) through small-diameter (and/or charged) nanopores are well-modeled as activated processes^53–56^ $\tau = \frac{h}{{k_{\mathrm{B}}T}}e^{{\Delta} G/k_{\mathrm{B}}T}$, where *τ* is the translocation time, ∆*G* is the height of the activation barrier, *k*_B_ is Boltzmann constant, *h* is the Planck constant, and *T* is the temperature of the system^53–55^. As the electric field *E*_0_ pulls the stalled DNA into the pore with a force *qE*_0_, where *q* is the effective charge of the molecule, translocation times decrease exponentially with the field in such activated entries, reducing the barrier by $W\sim {\int}_0^L {qE_0dz}$ associated to the work done by the applied field to move the DNA molecule a distance *L*^54,55^. Translocation times can then be written through $\tau = \tau _0e^{ - W/k_{\mathrm{B}}T},$ where *τ*_*0*_ is the zero-field translocation time, *k*_B_ is Boltzmann constant, and *T* is the temperature of the system^53–55^. In contrast, larger diameter nanopores with small surface charge densities only weakly interact with the translocating molecule and thus they do not exhibit activation barriers, leading to translocation times inversely proportional to the external electric fields, as the electrostatic forces balance with hydrodynamic drag^57–59^. For our Al_2_O_3_-coated PET nanopores, when the applied voltage was slightly increased from 400 to 600 mV, the average ssDNA translocation time increased five-fold, from 125 to 1217 ms (see Fig. 3), suggesting that field leakage increases the activation barrier for ssDNA, opposing translocation as illustrated in Fig. 2c.

**Fig. 4:**
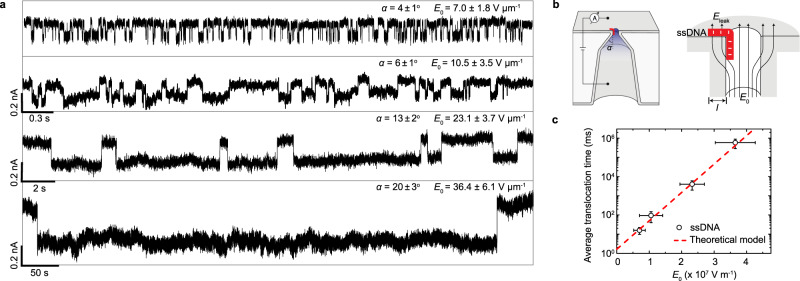
Modulation of translocation dynamics by angle control. **a** Representative current traces of 22 nt ssDNA translocating through four bullet-shaped Al_2_O_3_-coated (thickness, 3 nm) nanopores (diameter, 10 nm) with different half cone angles (*α*). The nanopores with larger cone angle allow more electric field (*E*_0_, as indicated) to be focused at the nanopore tip under the same applied voltage (500 mV) and thus higher magnitude of normal leakage field at the pore edge. With the increase of half cone angle and thus normal leakage field, the average translocation time can be increased exponentially from milliseconds to hundreds of seconds. **b** Left: schematic of the measurement apparatus using a bullet-shaped Al_2_O_3_-coated nanopores with half cone angles of *α*. Right: zoom in of the nanopore orifice with ssDNA electrostatically trapped at the pore edge by the normal leakage field. **c** The average translocation time dependence of *E*_0_ (*n* = 3). Error bars in the figure represent the standard deviation between independent experiments. The line represents the fit of the data to the theoretical model $\tau = \tau _0e^{ - \left( {{\Delta} W + {\Delta} W^ \ast } \right)/k_{\mathrm{B}}T}$, where *ΔW* and ΔW* are given by Eqs. (3) and (4), respectively, with *τ*_0_ = 1.595 ms, a charge per nt of 0.1e, and a length per nucleotide of 0.64 nm^62^.

To realize the full potential of field leakage induced retardation of ssDNA, we investigated different geometries to enhance field leakage. We use a series of bullet Al_2_O_3_-coated nanopores (diameter, 10 nm) with different half cone angles at their conical base. Nanopores with larger half cone angles *α* allow more electric field to be focused at the nanopore tip under the same applied voltage, as^51^

2 $$E_0\sim \frac{V}{{L^\prime }} + \frac{{Vtg\alpha }}{R},$$

with *L*′ the length of the nanopore (11.5 µm in all our experiments). Therefore, larger half cone angles allow higher magnitude of normal field leakage, according to Eq. (1). Such asymmetric nanopores with different half cone angles were fabricated by varying etching times after breakthrough (see Supplementary Fig. 3). Figure 4a compares representative current traces through four Al_2_O_3_-coated nanopores with half cone angles ranging from 4° to 20°. The magnitude of corresponding electric field *E*_0_ at the pore tip is indicated in Fig. 4a, while translocation times are presented in Fig. 4c. Strikingly, with the increase of *E*_0_ and thus normal leakage field, the average translocation time can be increased exponentially from tens of milliseconds to hundreds of seconds due to the activated nature of the entry.

**Fig. 5:**
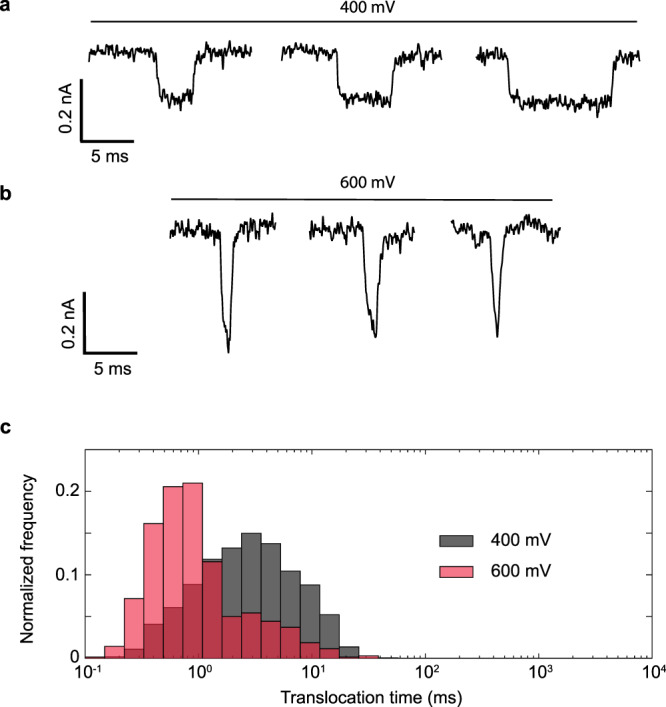
Effects of electric field leakage on dsDNA transport. **a** Representative translocation signals for 22 bp dsDNA translocations at applied voltages of 400 mV. **b** Representative translocation signals for 22 bp dsDNA translocations at applied voltages of 600 mV. **c** Normalized histogram of corresponding translocation times at applied voltages of 400 mV (*n* = 701 events) and 600 mV (*n* = 1762 events) and average translocation time as a function of applied voltage. Increasing the strength of electric field leakage decreases the translocation time of dsDNA. Data were acquired using a nanopore coated with 3 nm Al_2_O_3_ film (diameter, 8 nm; half cone angle, 9 ± 2°).

**Fig. 6:**
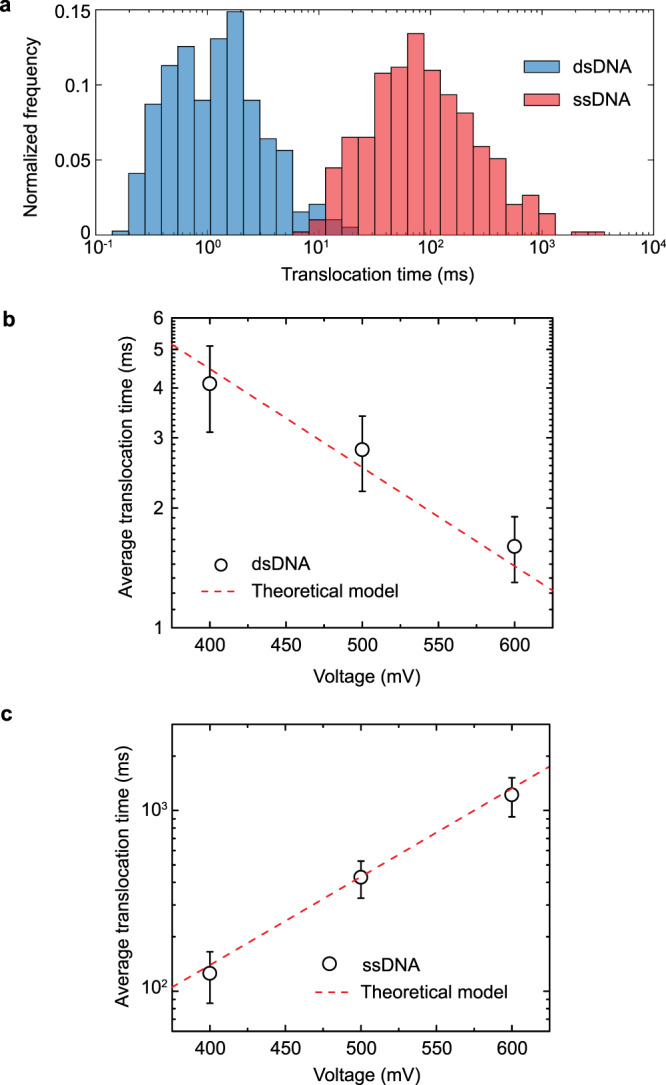
Comparison between dsDNA and ssDNA experimental translocation times. **a** Normalized histogram of translocation times for 22 nt ssDNA (*n* = 390 events) and 22 bp dsDNA (*n* = 492 events). ssDNA translocates much slower than dsDNA under the effect of electric field leakage (for a nanopore with a diameter of 10 nm, half cone angle ~7 ± 2° coated with 3 nm Al_2_O_3_ film under an applied voltage of 500 mV). These signature electrical signals allow discrimination (>97%) between ssDNA and dsDNA duplex translocation events (see Supplementary Fig. 6). **b** Average dsDNA translocation time dependence as a function of the applied voltage (*n* = 3). The line represents the fit of the data to the model ($\tau = \tau _0e^{ - W/k_{\mathrm{B}}T},$ where *W* is given by Eq. (3) with *τ*_0_ = 45 ms for a dsDNA with a charge per bp of 0.5e and a length per bp of 0.34 nm^70^). **c** Average ssDNA translocation time as a function of the applied voltage (*n* = 3). The line represents the fit of the data to the model ($\tau = \tau _0e^{ - \left( {W + W ^\ast } \right)/k_{\mathrm{B}}T}$, where *W* and W* are given by Eqs. (3) and (4), respectively, with *τ*_0_ = 1.595 ms, for a charge per nt of 0.1e and a length per nt of 0.64 nm). Error bars in the figures represent the standard deviation between independent experiments.

The high tunability of molecular pinning mechanism by varying the leakage field allows versatile control of translocation processes, which is difficult for other interactions^29^. It is expected that the charge, length, and mechanical properties of the translocating molecules can sensitively change the barrier and the translocation time, since the normal leakage field is confined to a film less than 3 nm in width. To test this selectivity, we analyzed translocation events of 22 base-pairs (bp) long double stranded DNA (dsDNA) molecules. The sample current traces for typical dsDNA translocation events and translocation time histograms at applied voltages of 400 and 600 mV are presented in Fig. 5. Interestingly, at both voltages, the translocation speed for dsDNA is observed to be orders of magnitude faster than that for the ssDNA. For example, at 400 mV, the average translocation time of dsDNA is around 4 ms, which is 2 orders of magnitude shorter than that of the ssDNA (and 2 orders of magnitude larger than reported translocation times in other nanopores^17,29^, see Supplementary Fig. 4). The translocation time of dsDNA has an opposite voltage-dependence to the ssDNA. Increasing the applied voltage from 400 to 600 mV, the average translocation time of dsDNA decreases from 4 to 1.5 ms, suggesting that the normal leakage field has much less effect on dsDNA translocation than on its ssDNA counterpart. With the opposite trends of ssDNA and dsDNA translocation times on voltage bias, the mean translocation times of the two molecules are about a factor of ~811 different at 600 mV and, taking into account the spread in their distributions, the probability of a ssDNA exhibiting the same translocation time as a dsDNA is less than 3% (Fig. 6a). Since an excess of ssDNA molecules with long translocation times will increase the assay time for a given number of translocation events, the selectivity gained at high field comes with a trade-off in longer assay time for ssDNA-rich mixtures (see Supplementary Fig. 5).

To explain these differences in translocation times, we may estimate the influence of field leakage on the activation barrier of the translocation events. When field leakage is involved, the driving field through the pore tip is reduced from *E*_0_ to $E_{{\mathrm{water}}}^{{\mathrm{normal}}}.$ Thus, there is a force $qE_{{\mathrm{water}}}^{{\mathrm{normal}}}$ which pulls the molecule into the pore, and one *qE*_leak_ that opposes it (see Fig. 2c). Assuming field leakage to be uniform for all base pairs outside the pore (and not to affect the bases inside it), these forces may be integrated from *z* = 0 (all bps outside) to *z* = *L* (all bps inside), leading to an estimate of the barrier reduction (see Supplementary Note 2)

3 $$W = Lq\left[ {E_{{\mathrm{water}}}^{{\mathrm{normal}}} - E_{{\mathrm{leak}}}/2} \right],$$

with *L* taken as the total length of the molecule (comparable to the sensing region of our pores). Decreasing the thickness *l* of the Al_2_O_3_ leads to higher field leakage and lower fields inside the pore, reducing *W* and leading to slower translocation events. For sufficiently long nanopores and small values of *l*, we may consider $E_{{\mathrm{leak}}} = E_{{\mathrm{water}}}^{{\mathrm{normal}}}\left( {\varepsilon _{{\mathrm{film}}}/\varepsilon _{{\mathrm{water}}}} \right)$, and Eq. (3) can be simplified to *W* = *LqE*_leak_[*ε*_water_/*ε*_film_ − 1/2]. Estimating *E*_leak_ from Eq. (1), we find excellent quantitative agreement to translocation time data for dsDNA if we reduce the relative permittivity of water *ε*_water_ from 80 to 6 (see Fig. 6b). This adjustment is reasonable, as the dielectric constant of surficial water layer of 2–3 molecules thick is known to be significantly smaller than that of bulk water (the literature value is 2–20^60,61^) because the rotational freedom of water dipoles decreases for the immobile layers near the surface. Thus, the normal field leakage *E*_leak_ near the pore edge surface becomes higher than normally expected because of the weak screening effect of the immobile water layers. A water permittivity comparable to the dielectric film would indeed produce a leakage field that is comparable to the applied field, according to the thin-film limit of Eq. (1).

As *ε*_water_/*ε*_film_ > 1/2, our previous derivation suggests that translocation time should still decrease exponentially with increasing voltage, even though the field leakage has increased the barrier as it reduces the pulling force. This is true for dsDNA. However, we found a curious opposite trend for the 22 nt ssDNA (see Fig. 6c), whose translocation time increased exponentially with the applied voltage. This suggests that other than adjusting for the total number of charges *q*, Eq. (3) needs to be modified for ssDNA. ssDNA molecules uncoil near hydrophobic surfaces, maximizing their hydrophobic interactions by means of increasing the contacts between the surface and their exposed aromatic rings^62^. In dsDNA molecules, this mechanism would require the breakage of the hydrogen bonds that stabilize the complementary base-pairs^63–65^. Therefore, hydrophobic interactions between dsDNA molecules and charged surfaces are limited to the two end base pairs, which have been suggested to present rapid (pico-seconds) opening and closing dynamics^66^. To cater to hydrophobic interactions present in ssDNA (and absent in their double stranded counterparts), a voltage-dependent term W* will be added to Eq. (3), which will model the van der Waals attraction of the hydrophobic rings of ssDNA towards the surface. We assume this term to be proportional to the field and a positive contribution to the barrier results,

4 $$W^ \ast = - kLqE_{{\mathrm{leak}}}$$

as field leakage brings the molecule into closer proximity to the membrane, stretching the molecule and thus increasing its affinity to the surface. Excellent agreement of our theory with experimental data is found by considering *k* ~ 0.9 (see Fig. 6c), suggesting hydrophobic interactions amplifies electrostatic pinning of ssDNA molecules to the pore surface, to the extent that physical adsorption occurs. The additional term in Eq. (4) only applies to ssDNA, leading to *W* + W* = *Lq E*_leak_ (*ε*_water_/*ε*_film_ − 1/2 − *k*) < 0 and a translocation time $\tau = \tau _0e^{ - \left( {W + W^ \ast } \right)/k_{\mathrm{B}}T}$ which increases with the applied voltage.

Our theoretical model has shown to work for short 22 nt DNA. However, this theory assumes all nucleotides in contact with the outer membrane feel the same leakage field. While this may be a good assumption for short molecules, it breaks down when *L* ≫ *l* because the leakage field is only localized at the pore orifice around the alumina layer and larger molecules will also feel more electrophoretic force that drives them into the pore. The pinning effect of the leakage field will hence be overwhelmed for long molecules. As a result, we expect faster translocation velocity for longer DNAs, which is consistent with our measurements shown in Supplementary Fig. 7. In other words, the field leakage effect is more effective for slowing down shorter ssDNA translocation. The normalized DNA velocity (nt ms^−1^) is observed to scale linearly with the DNA length since longer DNA carries more charge and thus experiences greater electrophoretic driving force for translocation. Nevertheless, the normalized translocation velocity for a 200 nt ssDNA translocating through the Al_2_O_3_-PET nanopore is around 50 nt ms^−1^, which is still much slower than the typical velocity (10^3^–10^5^ nt ms^−1^) in other solid-state nanopores (Supplementary Fig. 7).

**Fig. 7:**
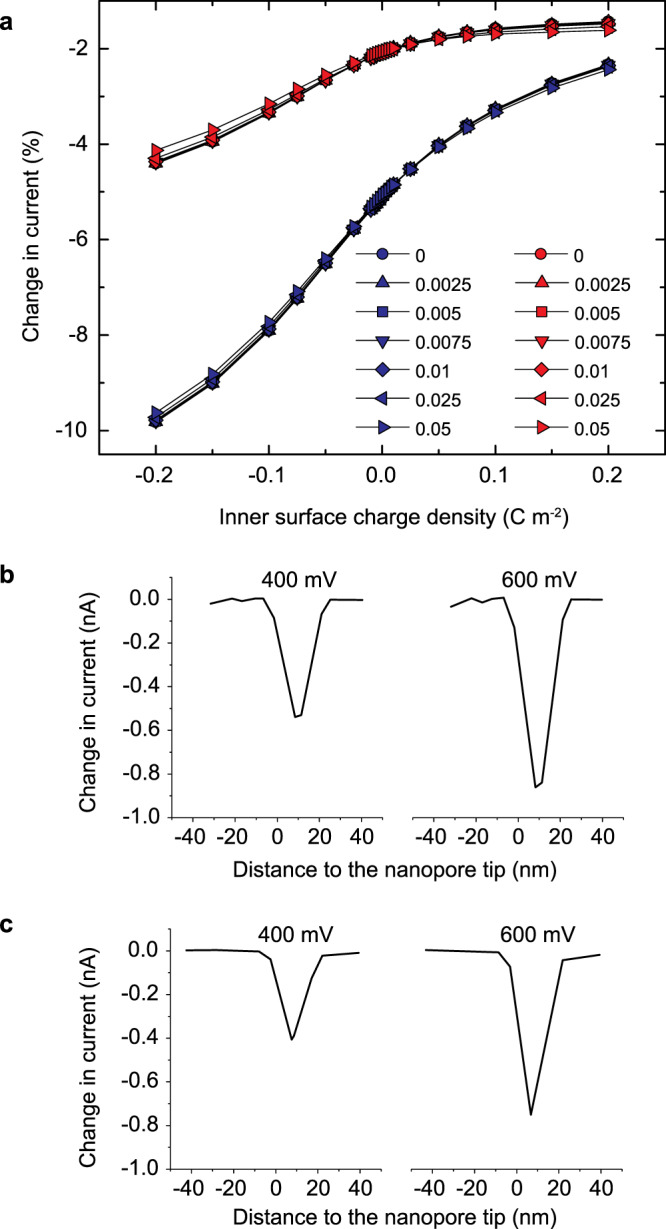
Finite-element-method simulation of the resistive signals. **a** FEM simulated normalized change in current for a dsDNA molecule (blue) and a ssDNA molecule (red) translocating through the axis of a nanopore in function of the inner (horizontal axis) and outer (symbol shape) surface charge density at 500 mV. Note that dsDNA molecules always lead to higher current drops than their single stranded counterparts, as they have higher cross-section areas. **b** FEM simulated current drops for single stranded molecules translocating through the walls of the nanopore at 400 and 600 mV. **c** FEM simulated current drops for double stranded molecules translocating through the axis of the nanopore at 400 and 600 mV. Note that ssDNA molecules translocating through the pore walls present comparable resistive signals with their double stranded counterparts translocating through the bulk of the nanopore, in agreement with Figs. 3 and 5. Simulation details are presented in Supplementary Note 3.

This adsorption of ssDNA molecules to the pore surface is further confirmed by analyzing the current change associated to the translocation events. Due to their smaller cross-section area, single stranded molecules block less current than their double stranded counterparts, leading to lower current drops at high salt concentrations (such as the ones used in our experiments)^67,68^ (see Fig. 7a). However, we observe comparable, if not higher, current drops for our single stranded molecule experiments (see Figs. 3 and 5), which can only result from significant modulation of the ionic conductivity due to surface effects^62^. In conical and bullet-shaped nanopores, the sensing area is localized near the tip, where the field is focused^51^ and the surface charge of the thin alumina coating controls the ionic conductance in this key region. Adsorption of molecules near this region will then change the effective surface charge density of the pore walls (lowering it in our case, as Al_2_O_3_ is positively charged), depleting their counter-ions and leading to lower current. This would then lead to much higher current drops than for molecules translocating far from the pore walls^62^ (see Fig. 7b). We have reproduced the resistive current pulses of 22 nt ssDNA and 22 bp dsDNA by FEM simulation (Fig. 7b, c), with comparable amplitudes for both due to adsorption of ssDNA.

**Fig. 8:**
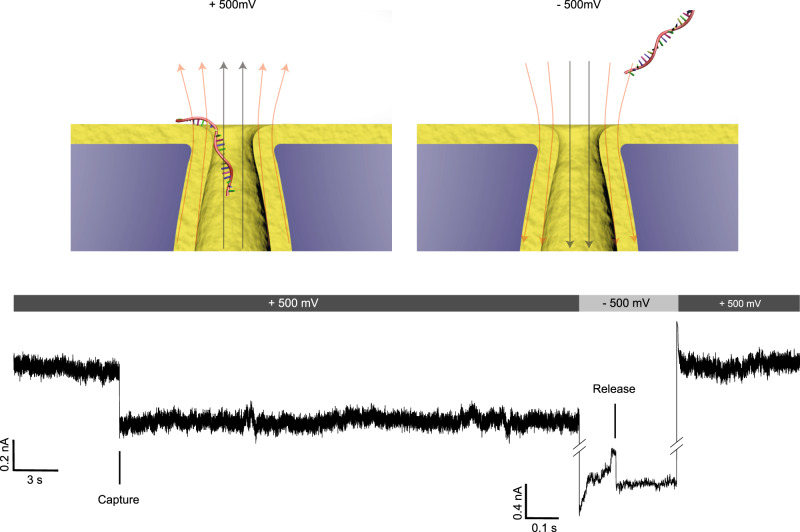
Reversibility of the molecular pinning. Electrical recording of 22 nt ssDNA pinning at the pore edge of a bullet-shaped Al_2_O_3_-coated (thickness, 3 nm) nanopores (diameter, 10 nm) with a half cone angles of 20 ± 3° under applied voltage of +500 mV (energy barrier, 12.7 *kT*) and ssDNA escaping from the nanopore after the electrostatic trapping effect is switched off by reversing the polarity of applied voltage to −500 mV.

Finally, when the applied field is *E*_0_ = 36.4 ± 6.1 V μm^−1^, the average translocation time of ssDNA can last several minutes (Fig. 4a). Even under such strong normal leakage field, this electrostatic trapping effect is completely reversible. This is illustrated in Fig. 8, where the current recovered to the base level after a negative voltage was applied. Once the direction of applied voltage and normal leakage field is reversed, the electrostatic trapping effect is switched off, and ssDNA can escape from the nanopore (see also Supplementary Fig. 8). This reversible electrostatic trapping effect reduces the possibility of permanent nanopore blocking by translocating molecules, a common issue for nanopore sensors^69^. It is worth noting that the observed long translocation time does not imply that every base translocates through the nanopore at the same slow velocity. The translocation kinetics through the Al_2_O_3_-PET nanopores resemble a “stick-slip” motion in which the DNA rapidly translocates as soon as the applied force overcomes the energy barrier. A stick-slip translocation mechanism may give long dwell times for only a single segment along a DNA, which is not helpful for resolving the other segments and unfavorable in nanopore-sequencing technology. On the other hand, the high translocation speed of DNA is probably not the biggest problem for sequencing with solid-state nanopores. The main problem is that the ionic current signal in solid-state nanopores does not provide the resolution for base determination.

## Discussion

We have designed dielectric film coated solid-state nanopores in insulating polymer membranes, with an enhanced leakage field at the pore edge that can delay the translocation of ssDNA molecules by 5 orders of magnitude, thus providing a practical method to achieve up to 5 orders of temporal resolution enhancement for sensing applications. This large range of translocation times is due to an activated transport mechanism into the pore endowed by the pinning field, which can lead to actual adsorption, with an exponential dependence on the applied field and a barrier height that is sensitive to the affinity of the molecule to the surface. The prolonged molecular-pinning time allows short nucleic acids to produce observable and distinct resistive signals and yet does not clog the nanopores or interfere with the resistive signal current. The reported enhanced sensitivity and selectivity would be useful for multiplex profiling of target microRNAs after hybridizing them with designed bar-coded oligos with dangling tails of different signature translocation times within the large range reported here. That the pinned ssDNA actually absorbs onto the edge suggests specific sequences or protein attachments can increase the library volume. Further studies of the interactions between DNA and normal field leakage in the context of voltage-driven DNA translocation may allow DNA translocate through the nanopore base-by-base, enabling a more controlled transport through nanopores equipped with transverse electrodes and allowing high-resolution sequencing or DNA/protein interaction analyses.

## Methods

### Fabrication of Al_2_O_3_-coated polymeric nanopore

The 12 μm thick PET foils were irradiated with single swift heavy ions (Au) with energy of 11.4 MeV per nucleon at the GSI in Darmstadt, Germany. An irradiated foil was subsequently etched at room temperature (295 K) by an asymmetric etching method, where the foil was mounted between two isolated containers that contained an etchant solution of 2.5 M NaOH in 1:1 MeOH/H_2_O, a stopping solution of 1 M HCOOH, and 1 M KCl aqueous solution, respectively. The etching process started from one side of the PET foil, but was immediately stopped when etched through, and as a result, a single trumpet-like nanopore was formed on each irradiated PET foil. Bullet-shaped nanopores with different half cone angles were fabricated by tuning etching times after breakthrough (see Supplementary Fig. 3). A secondary symmetric etching process (2 M NaOH) was applied to enlarge the tip size. In all cases, the radius of the base was around 500 ± 80 nm, as determined by electron microscopy. The final tip radius was determined by a conductance measurement. Thermal ALD Al_2_O_3_ films of 3 or 10 nm were grown in a commercial (Cambridge Nanotech, Savannah S100) ALD reactor using trimethylaluminium (TMA) and de-ionized (DI) water as precursors. Due to the self-limiting nature of the ALD surface chemistry reactions, the film thickness was precisely controlled by setting a certain number of the ALD cycles. An ALD growth cycle of Al_2_O_3_ deposition consists of the following steps and parameters: TMA pulse 0.02 s, N_2_ purge 15 s, H_2_O pulse 0.02 s, N_2_ purge 20 s. A low deposition temperature of 110 °C was chosen to prevent thermal damage to the polymer PET.

### Experiments of DNA transport

A PET foil with a single Al_2_O_3_-coated polymeric nanopore was mounted between two isolated channels that were both filled with buffered 1 M KCl aqueous solution (0.01 × PBS, pH = 7.4). A patch clamp amplifier (Axopatch 200B, Molecular Devices Inc.) with Ag/AgCl electrodes was used to measure the current trace and the current−voltage response across the nanopore. The polarity of the applied voltage was referenced to the tip side electrode. The current data were collected at 50 or 100 kHz with a low-pass Bessel filter of 10 kHz. For the DNA transport experiment, the buffered 10 pM 22 nt ssDNA and 22 bp dsDNA (Integrated DNA Technologies) solution (in 1 M KCl, 0.01× PBS, pH = 7.4) was always freshly made prior to each experiment and was injected to the tip side of the nanopore; 22 bp dsDNA was obtained by hybridizing two complementary oligos and then purified by gel electrophoresis. Unless otherwise specified, a positive voltage of 500 mV was used in the transport experiment to drive the negatively charged molecules through the nanopore from tip to base.

### Finite-element-method simulations

All FEM simulations were performed with the commercial code COMSOL. Simulation details of the field leakage are presented in Supplementary Note 1. Simulations of the translocation events were performed following a methodology previously published^62^. The 2D simulations were performed for the axisymmetric systems (bulk translocations) while 3D simulations were performed for the non-symmetric systems (adsorption). For each simulation, an initial mesh heavily refined on narrow regions, near the translocating agents, and near charged surfaces was considered. This mesh was refined through 5 mesh adaptation steps and convergence of the current was assessed during these adaptations (see Supplementary Note 3).

## Supplementary information

Supplementary Information

## Data Availability

Data supporting the findings of this paper are available from the corresponding authors upon reasonable request. The source data underlying Figs. 1b–d, 2, 3, 4a, c, 5, 6, 7, 8 and Supplementary Figs. 2, 3, 4, 5, 6, 7, 8, 12, 13, and 14 are provided as a Source Data file (10.6084/m9.figshare.13238687.v1).

## Code availability

Code for data analysis is available from the corresponding author upon reasonable request.
