# Supplementary material for: Slowing down DNA translocation through solid-state nanopores by edge-field leakage
Source: Nat Commun. 2021 Jan 8;12:140. doi: 10.1038/s41467-020-20409-4 (PMC7794543; doi:10.1038/s41467-020-20409-4)
Supplement: Supplementary file 1 — Supplementary Information [file 41467_2020_20409_MOESM1_ESM.pdf]

Supplementary Information

Slowing Down DNA Translocation through Solid-State Nanopores by Edge-Field Leakage

Ceming Wang[1], Sebastian Sensale[2], Zehao Pan[1], Satyajyoti Senapati[1] and Hsueh-Chia Chang[1, 2]*

[1] Department of Chemical and Biomolecular Engineering, University of Notre Dame, USA

[2] Department of Aerospace and Mechanical Engineering, University of Notre Dame, USA

* Email: hchang@nd.edu

This file contains:

- Supplementary Figure 1
- Supplementary Figure 2
- Supplementary Figure 3
- Supplementary Figure 4
- Supplementary Figure 5
- Supplementary Figure 6
- Supplementary Figure 7
- Supplementary Figure 8
- Supplementary Figure 9
- Supplementary Figure 10
- Supplementary Figure 11
- Supplementary Figure 12
- Supplementary Figure 13
- Supplementary Figure 14
- Supplementary Note 1
- Supplementary Note 2
- Supplementary Note 3
- Supplementary References 1-4

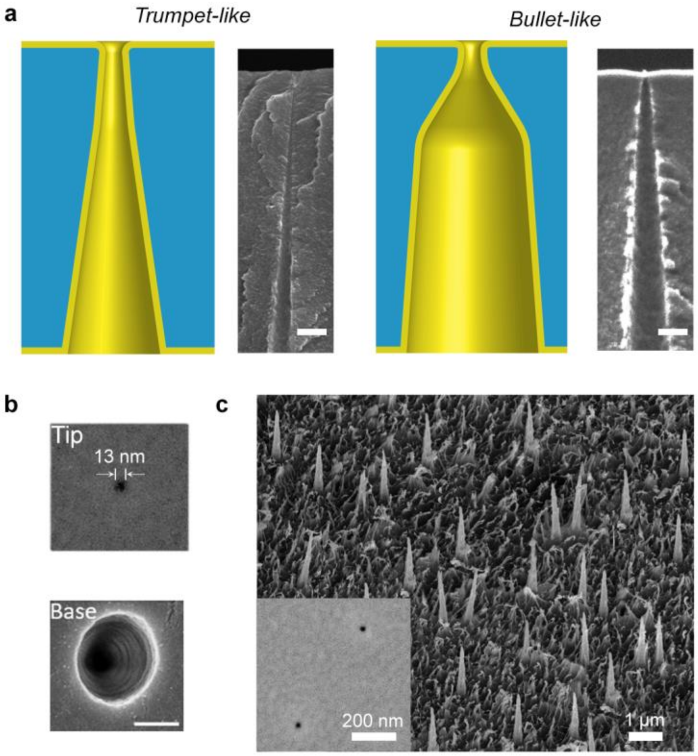

Supplementary Figure 1 SEM characterization of the as-prepared nanopores. a Schematic illustration (not to scale) and cross-sectional SEM images of a trumpet-like nanopore (left) after etching breakthrough and a bullet-like nanopore (right) after extended etching. Scale bar = 400 nm. **b** Representative SEM images of the tip side (top) and the base side (bottom) of an ALD Al_2O_3 coated polymeric nanopore. Scale bar = 500 nm. **c** SEM images of conical Al_2O_3 replicas obtained by coating the conical nanopore membrane with 200 cycles of ALD Al_2O_3 and then treating with RIE to selectively remove the PET polymer matrix. Inset: SEM image of the pore tip side before RIE etching.

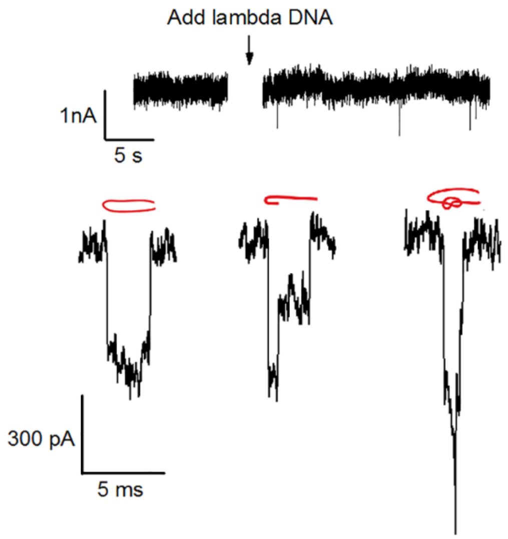

Supplementary Figure 2 Detection of Lambda DNA using bare PET nanopore. Representative current traces for lambda DNA (48.5 kbp) translocation through a bare bullet-shaped PET nanopore without Al_2O_3 film coating. A series of multiple blockade levels events were observed, indicating lambda DNA with various folded configurations translocating through the pore. A close-up view of the three characteristic events are displayed, including fully folded, partial folded, and fully folded containing a knot. Similar characteristic translocation signals obtained with other solid-state nanopores have been reported in previous literatures. The average translocation time for lambda DNA in our study is around 2.6 ms, corresponding to a translocation velocity of 54 ns per base. Thus, if a 22 nt ssDNA also translocates through the uncoated nanopore at the same velocity, the translocation time would be only 1.2 µs, which is undetectable as shown in Figure 2a given the typical sampling frequency of 100 kHz in our nanopore sensing experiment. The uncoated PET nanopores have a bullet-like shapes (half cone angle ~ 7 ± 2°) and tip size of 35 nm. The applied voltage is 500 mV.

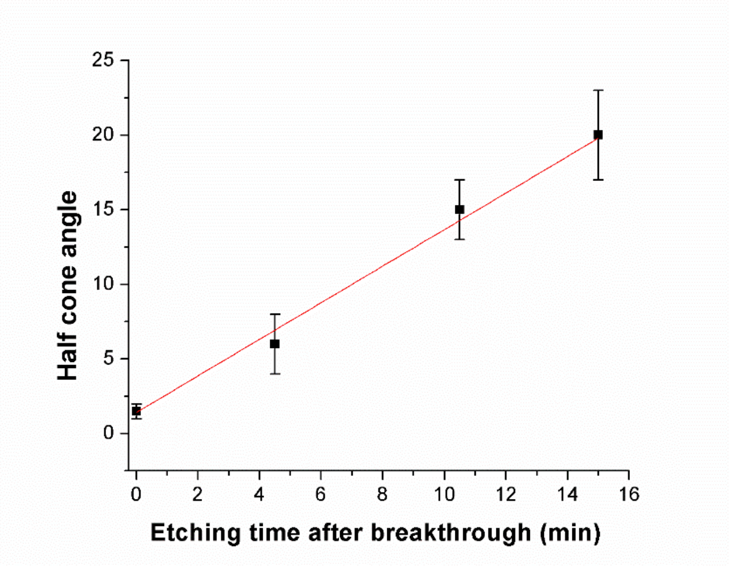

Supplementary Figure 3 Tuning the half cone angle of the PET nanopores. Measured half cone angle of the asymmetric PET nanopore as a function of the time of etching under asymmetric conditions after breakthrough. Error bars represented the standard deviation between different nanopores. The pore geometry evolves through a variety of configurations (half cone angles) with advancing time after breakthrough. While immediately after breakthrough the pore tips are trumpet-shaped, further etching is strongly affected by osmotic effects which eventually lead to bullet-shaped pore tips (Supplementary Reference 1). Thus, asymmetric nanopores with different half cone angles can be fabricated by varying etching times after breakthrough.

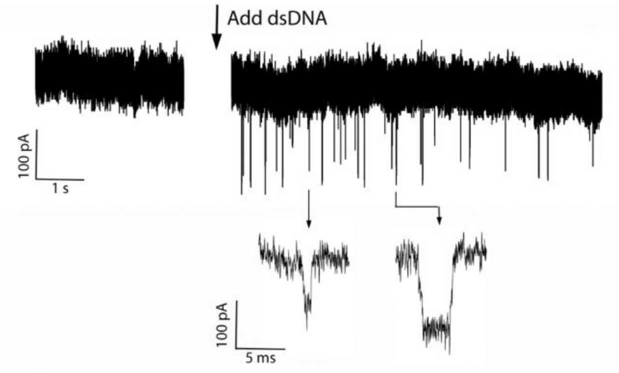

Nanopore	Translocation time (ms)	dsDNA length	Translocation speed	Reference
Graphene-Al_2O_3-Graphene	0.34	850bp dsDNA	0.4 μs bp^{-1}	Supplementary Reference 2
SiN nanopore	0.02	25bp dsDNA	0.8 μs bp^{-1}	Supplementary Reference 3
Al_2O_3-PET nanopore	2.7	22bp dsDNA	123 μs bp^{-1}	This study

Supplementary Figure 4 Slow dsDNA translocation in the Al_2O_3-PET nanopores. Top: example current recordings of 22 bp dsDNA translocation through bullet-shaped PET nanopores (10 nm, half cone angle ~ 7 ± 2°) coated with 3 nm Al_2O_3 film under an applied voltage of 500 mV. Bottom: comparison between different solid-state nanopore platforms for short dsDNA translocation. Although this normal leakage field induced electrostatic interaction with dsDNA is not as strong as that for its ssDNA counterpart, the translocation of short dsDNA through our nanopores with the presence of normal field leakage is still much slower than that for other solid-state nanopores.

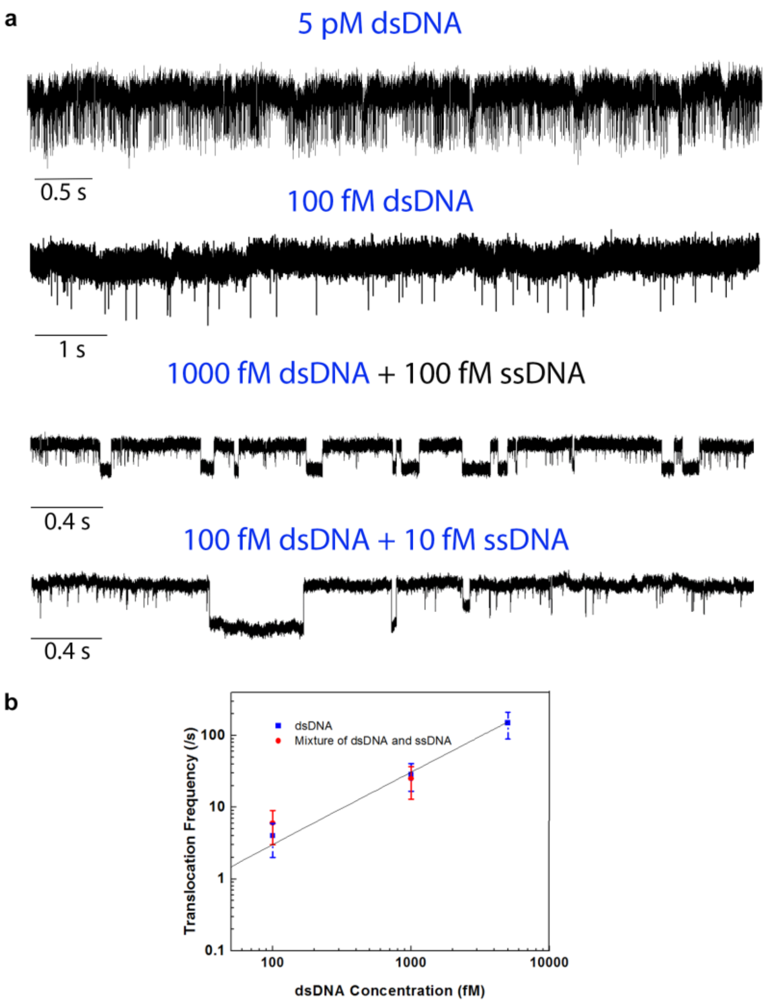

Supplementary Figure 5 ssDNA-dsDNA mixture translocation frequency. a. Example current recordings for the spiked pure dsDNA samples or mixed with ssDNA with concentrations as indicated. The bullet-shaped nanopores (10 nm) is coated with 3 nm Al_2O_3 film under an applied voltage of 500 mV. **b.** The translocation frequency as a function of dsDNA concentration. Error bars represented the standard deviation between independent experiments. It is worth noting that this highly selective sensing does not come with a tradeoff in throughput as long as the ssDNA in the sample is below 10%. When the ssDNA in the sample is above 10%, their long translocation may start to reduce the throughput. The frequency of the current blockage events observed increased linearly with the DNA concentration in the bulk solution, indicating these current blockages are due to different DNA molecules translocating through the pore. Such linear dependence would not occur if the current blockage events were due to the fluctuations of a DNA molecule in different locations.

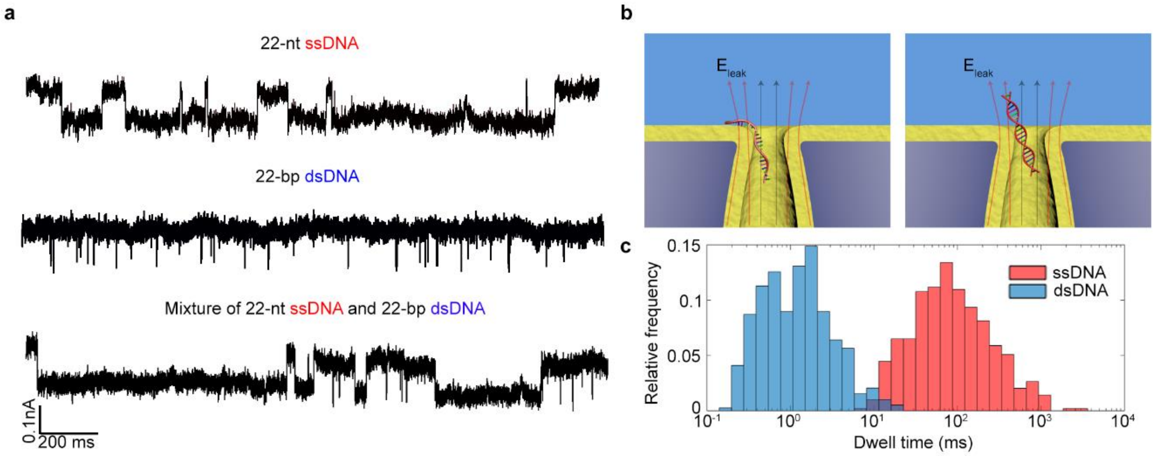

Supplementary Figure 6 Short ssDNA and dsDNA discrimination. a Representative current traces for 22 nt ssDNA, 22 bp dsDNA, and the mixture of 22 nt ssDNA and 22 bp dsDNA (1:1) translocation through bullet-shaped nanopores (10 nm, half cone angle ~ 7 ± 2º) coated with 3 nm Al_2O_3 film under an applied voltage of 500 mV. **b** Schematic of 22 nt ssDNA and 22 bp dsDNA translocation under the effect of electric field leakage. ssDNA molecules (persistence length, 2 nm) can easily deform and be pinned at the pore edge. The stiffer dsDNA molecule (persistence length, 50 nm) tends to be linearly oriented under the influence of the strong local electric field and could be expected to have weak interactions with the leakage field. **c** Normalized histogram of translocation times for 22 nt ssDNA and 22 bp dsDNA. ssDNA translocates much slower than dsDNA under the effect of electric field leakage. For a nanopore with intermediate field leakage. These signature electrical signals allow discrimination (>97%) between ssDNA and dsDNA duplex translocation events.

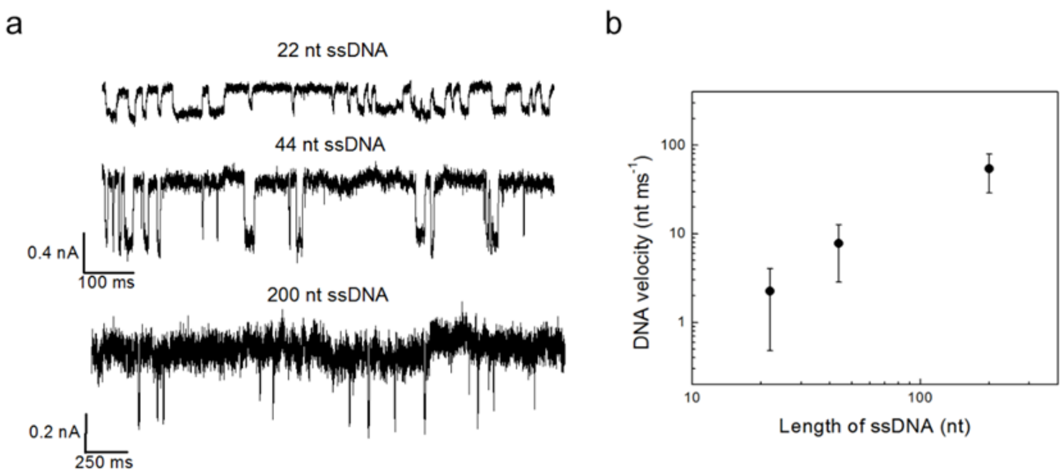

Supplementary Figure 7 Example translocation current traces of ssDNA with different lengths. a Representative current traces for ssDNA with different lengths (22 nt, 44 nt, and 200 nt) translocation through a bullet-shaped PET nanopore with 3 nm Al_2O_3 film coating. The Al_2O_3-PET nanopores have a bullet-like shapes (half cone angle ~ 5 ± 1º) and pore tip size of 10 nm (22 nt and 44 nt) or 35 nm (200 nt). The applied voltage is 500 mV. **b** The DNA velocity versus the length of ssDNA. Error bars represented the standard deviation between independent measurements.

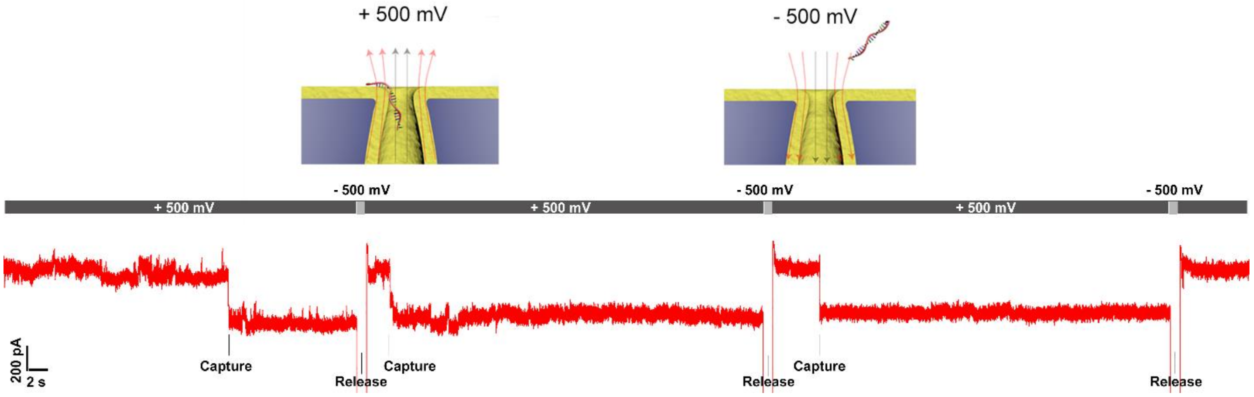

Supplementary Figure 8 The reversible electrostatic trapping effect. Continuous electrical recording of 22 nt ssDNA pinning at the pore edge under applied voltage of + 500 mV (energy barrier, 12.7 kT) and ssDNA escaping from the nanopore after the electrostatic trapping effect is switched off by reversing the polarity of applied voltage to – 500 mV. This electrostatic trapping effect is completely reversible. Data was obtained using a bullet-shaped Al_2O_3–coated (thickness, 3 nm) nanopores (diameter, 10 nm) with a half cone angle of 20 ± 3°.

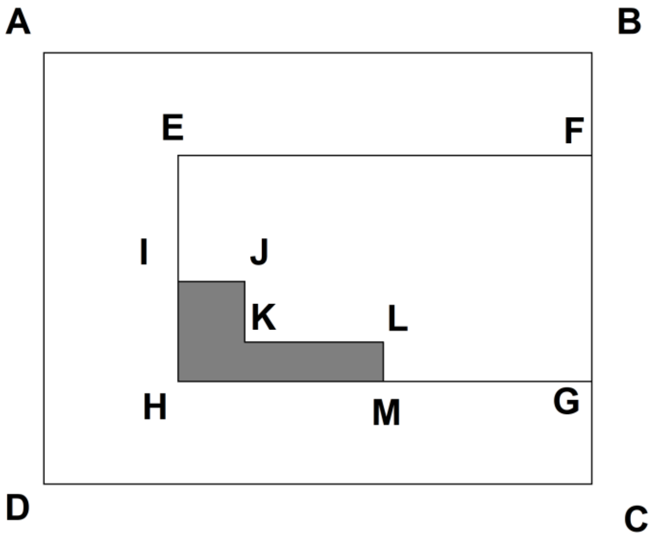

Supplementary Figure 9 Schematic of the simulated nanopore.

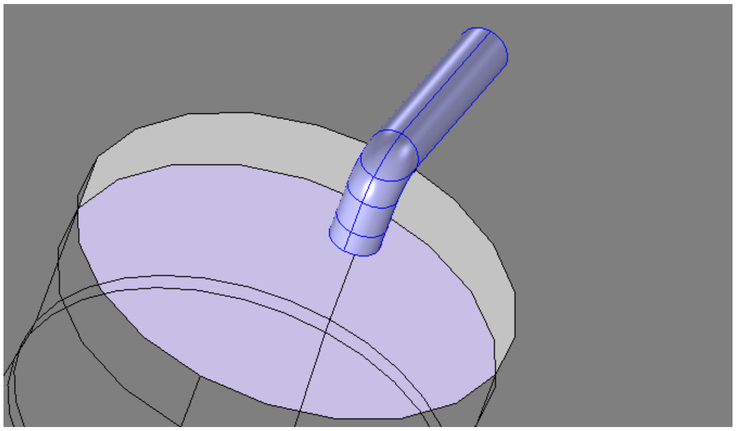

Supplementary Figure 10 FEM image of the translocation of a ssDNA molecule through a nanopore.

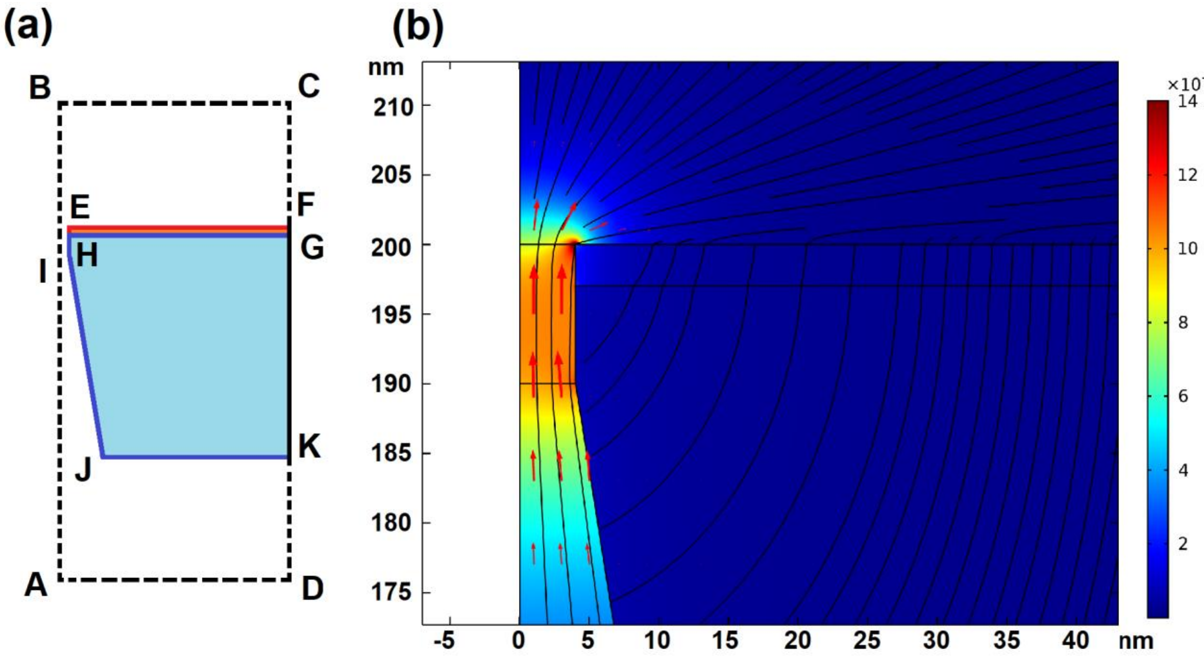

Supplementary Figure 11 a Sketch of the axisymmetric geometry used in the simulation. **b** Electric displacement field ($\mathrm{C\,m^{-2}}$) obtained from FEM simulations with $\varepsilon_{\mathrm{f}} = 80, \varepsilon_{\mathrm{m}} = 3, \varepsilon_{\mathrm{film}} = 8, V_0 = 0.5\ \mathrm{V}, c_0 = 1\mathrm{M},\ \ r_{\mathrm{p}} = 4\mathrm{nm}\ ,\ \ l = 3\mathrm{nm}, L_{\mathrm{p}} = 7\mathrm{nm}, L = 100\mathrm{nm}, \rho = 1000\mathrm{kg/m^3}, \mu = 0.0024\mathrm{Pa\ s}\ ,\ \ z_+ = z_- = 1\ ,\ \ D_+ = D_- = 2\times10^{-9} m^2\, s^{-1}$. The surface plot represents the norm of the electric displacement while arrows and streamlines correspond to its vectoral form. Segments AB, BC, CF and DK measure 300 nm, 100 nm, 100 nm and 100 nm, respectively.

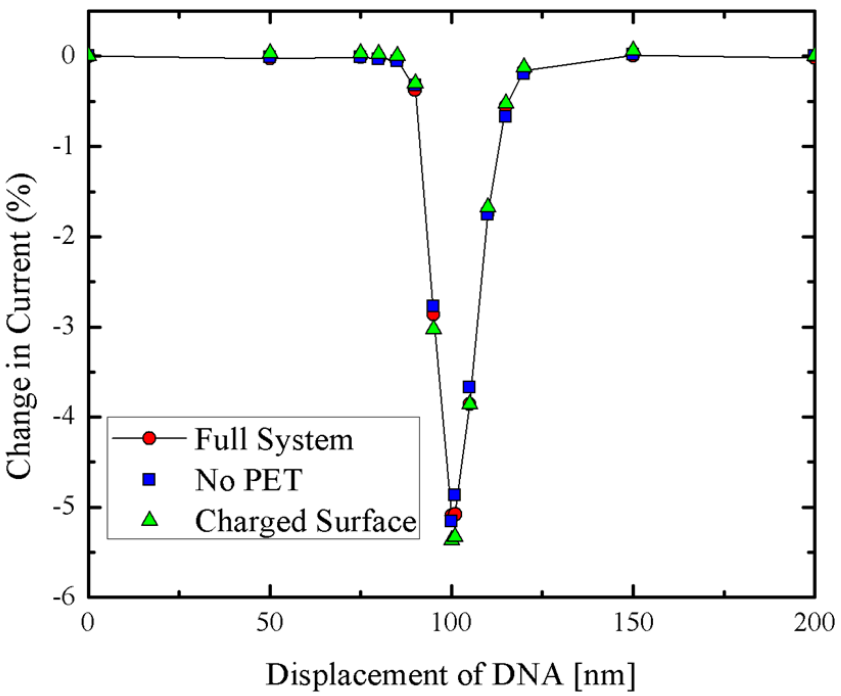

Supplementary Figure 12 Normalized change in current ($\chi = (I - I_0)/I_O$) for a dsDNA molecule with different conditions of the dielectric materials, measured from B.

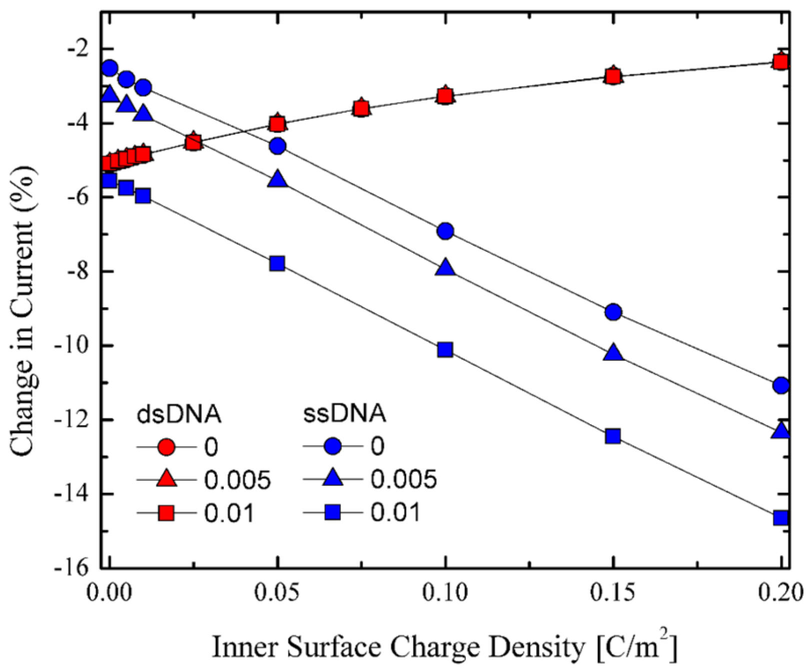

Supplementary Figure 13 Normalized current drops for different inner (horizontal) and outer (symbol) surface charge densities for a dsDNA (red) and a ssDNA (blue) molecule, translocating through the middle (dsDNA) and surface (ssDNA) of the pore, respectively.

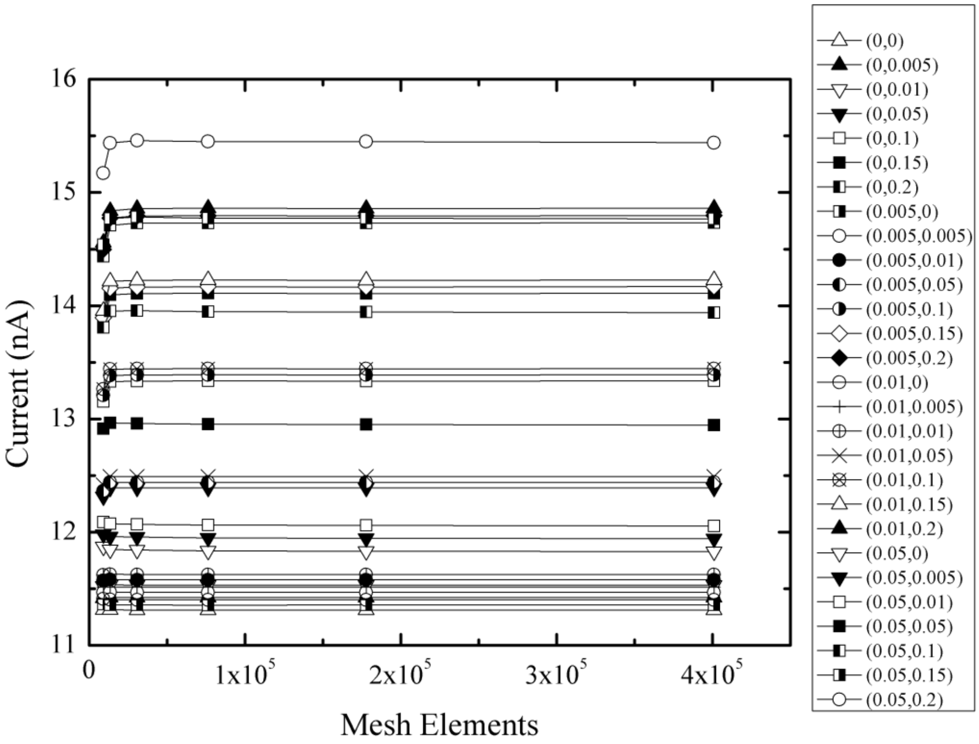

Supplementary Figure 14 Convergence studies for the baseline at 0.5 V for different pairs of (outer surface charge density, inner surface charge density).

Supplementary Note 1

Estimation of normal leakage field. We can estimate the normal field leakage through the high-permittivity dielectric film on a cylindrical insulating polymer nanopore orifice, which models the straight section at the tip of the bullet nanopore. The tangential field is dominant in this region due to the slenderness of the orifice. Consider a Gauss volume with radius *R+l* and length *L'*>> *(R+l)* that is placed coaxially with the straight tip, with the end surface on the outside of the polymer pore where the field is leaked. At the other end on the base side, the electric field E_0 is entirely tangential to the pore wall and is confined to the water phase within the pore. (This field represents the focused field by the conic base). The field lines then enter the dielectric film but do not penetrate into the polymer within the control volume. Hence, the electric flux balance according to the Gauss divergence theorem stipulates that

$$\varepsilon_{\mathrm{water}} E_0 \cdot \pi R^2 = \varepsilon_{\mathrm{water}} E_{\mathrm{water}}^{\mathrm{normal}} \cdot \pi R^2 + \varepsilon_{\mathrm{film}} E_{\mathrm{film}}^{\mathrm{normal}} \cdot \pi((R+l)^2 - R^2) \quad (1)$$

where *R* is the radius of nanopore orifice, *l* is the thickness of the dielectric film, and the two electric fields are normal fields exiting the tip end of the Gauss volume from the liquid and dielectric film, respectively. To leading order, the electric field in water and dielectric film are mostly tangential and normal to the exit surface of the Gauss volume. As both normal fields at the exit surface are tangential fields within the Gauss volume, they should be equal for a sufficiently long pore and we can estimate both by

$$E_{\mathrm{film}}^{\mathrm{normal}} = \frac{\varepsilon_{\mathrm{water}} E_0 R^2}{\varepsilon_{\mathrm{water}} R^2 + \varepsilon_{\mathrm{film}}((R+l)^2 - R^2)} \quad (2)$$

We have numerically solved the Laplace equation for a high-permittivity dielectric film on an insulating polymer nanopore orifice geometry using COMSOL Multiphysics v3.5a. Figure 1c shows collapse of numerical normal field data by Eq. (2) for different film permittivities (5-17) and film thicknesses (1-27 nm). This normal field around the tip penetrates across the dielectric film into the water bulk with a field E_{leak} governed by displacement continuity,

$$E_{\mathrm{leak}} = (\varepsilon_{\mathrm{film}}/\varepsilon_{\mathrm{water}}) E_{\mathrm{film}}^{\mathrm{normal}} = \frac{\varepsilon_{\mathrm{film}} E_0 R^2}{\varepsilon_{\mathrm{water}} R^2 + \varepsilon_{\mathrm{film}}((R+l)^2 - R^2)} \quad (3)$$

It is important to note that Eq. (2) was calculated by considering $L' >> (R+l)$, and the field lines may enter the dielectric material in a different manner if those magnitudes are comparable.

Finite-element-method simulations were performed using COMSOL v3.4. An axisymmetric simulation box with a height of 300 nm and a width of 500 nm was considered. In this box, our nanopore is considered as a region of radius R and height 100nm occupying the middle 1/3 of the simulation box as shown in Supplementary Figure 9. A high permittivity region HIJKLMH is considered, with permittivity $\varepsilon_{\mathrm{film}}$. Region ABFEIGMGCDA has a permittivity $\varepsilon_{\mathrm{leak}}$, while region EFGMLKJIE is considered as vacuum. Segments IJ and LM have a thickness l, while segments JK and KL have a thickness of 10 nm. The COMSOL module of electrostatic is used, with AB taken at a constant voltage of 2V and CD taken as ground. No normal electric field boundary conditions (BCs) are imposed on segments EF, FG, GM, ML, LK, KJ, JI, while continuity of the electric field is imposed on segments IH and HM. Axial symmetry is imposed on AD, and no normal electric field on BF and GC. A mesh refined near the dielectric material is considered and the Laplace equation is solved in the whole domain (minus the vacuum region EFGMLKJIE).

Supplementary Note 2

Derivation of the theoretical activation barrier. Consider a DNA molecule found near the top membrane of a nanopore, as shown in Supplementary Figure 10. Due to the applied voltage, the force that pulls the DNA molecule into the pore may be estimated through $F = qE_{\mathrm{water}}^{\mathrm{normal}}$, where q is the net charge of the molecule. Field leakage only affects those molecules in contact with the outer membrane and thus we may estimate the force resulting from leakage through $F = pE_{\mathrm{leak}}$, where p is the net charge outside the nanopore. We then have a net force in the molecule given by

$$F = qE_{\mathrm{water}}^{\mathrm{normal}} - pE_{\mathrm{leak}}.$$

We may estimate p through $p = \frac{q}{L}(L - x)$, where L is the length of the molecule and x the position of the tip of the molecule with respect to the tip of the nanopore (at $x = 0$, the molecule is completely outside the pore, while at $x = L$ all of its atoms are inside). The work done by these forces may then be estimated through

$$W = \int_0^L qE_{\mathrm{water}}^{\mathrm{normal}} - \frac{q}{L}(L - z)E_{\mathrm{leak}}\, dz = qLE_{\mathrm{water}}^{\mathrm{normal}} - \frac{qL}{2}E_{\mathrm{leak}}. \quad (4)$$

It is important to note that this theory assumes all nucleotides in contact with the outer membrane feel the same effect of field leakage. While this may be a good assumption for short molecules, it breaks down when $L \gg l$ and thus larger molecules will feel less of an effect of field leakage, the force that pulls DNA into the pore will dominate, and we will observe faster translocation events.

Supplementary Note 3

Finite-Element-Method simulation of the translocation event. Supplementary Figure 11 depicts a conical nanopore embedded within a dielectric membrane with relative permittivity ε_{m}, which connects two identical reservoirs on either side. On the upper side of the membrane, a high permittivity dielectric coating of thickness l and relative permittivity $\varepsilon_{\mathrm{film}}$ is considered. The computational domain includes the fluid domain Ω_{f}, the high permittivity coating Ω_{film}, and the membrane domain Ω_{m}, which are enclosed by segments ACBFEHIJKD, EFGH and GHIJK respectively. The nanopore consists on a cylindric portion (EHI) of length $l + L_{\mathrm{p}}$ and radius r_{p}, and a conical portion (IJ) of semi-angle θ and length $L - (l + L_{\mathrm{p}})$. The length and radius of the reservoirs are sufficiently large to ensure the numerical results are independent of the reservoir size. The two reservoirs are filled with a 1-1 symmetric electrolyte solution containing ions with equal mobility, at the bulk ionic concentration c_0, density ρ, dynamic viscosity μ, and relative permittivity ε_{f}. An electric potential difference V_0 is applied between the bottom (AD) and top (BC) of the reservoirs to generate the ionic current and electroosmotic flow through the nanopore.

The strongly coupled Poisson-Nernst-Planck and Navier-Stokes equations are solved for this axisymmetric geometry with the commercial finite-element software COMSOL 5.3. An exhaustive study of the proper meshing is performed to ensure the numerical results are independent of the meshing. Finer meshing is generated in the region close to the tip of the nanopore and near the boundaries of the high-permittivity coating.

As the Reynolds number is usually small in nanofluidic devices, the inertial term in the Navier-Stokes equations is neglected. The boundary conditions for the fluid motion are as follows: no-slip boundary conditions on FEHIJK, axial symmetry along segment AB, normal flow with pressure $p = 0$ on segments BC and AD and slip boundary conditions for segments CF and KD. Concerning the electric potential, axial symmetry is imposed on AB while zero normal electric field is imposed on CFGKD. As segments EF, EH, HI, IJ and JK are the walls of the reservoirs and the nanopore, no ion flux is imposed on those boundaries. Segments AD and BC are far from the nanopore and thus the ionic concentrations at both boundaries are the bulk concentrations c_0. Axial symmetry is imposed on AB, and zero normal ionic flux is applied along CF and KD since they are in the bulk electrolyte reservoirs.

Supplementary Figure 12 presents the electric displacement field $\vec{\mathbf{D}} = \varepsilon\vec{\mathbf{E}}$ obtained from FEM simulations at the tip of the nanopore. Note that a large field penetration exists near the tip of the nanopore even for low permittivity dielectric materials. Neither ions nor the fluid penetrates through the wall of the nanopore. For a positive V_0, the penetrating field induces a positive surface charge density to the outer surface EF and a negative one to the inner surface of the nanopore. The (absolute) value of this induced surface charge density increases with voltage, leading to a net voltage-dependent surface charge density on the membrane, implying higher interactions of the translocating molecules with the outer surface at higher voltages.

To characterize the current drop during a translocation event, finite element method simulations of the translocation of double and single stranded molecules through the middle of the nanopore were performed. A 22-base-pairs-long dsDNA molecule and a 22-nucleotides-long ssDNA molecule were considered. The dsDNA molecule was modeled as a cylinder with radius $r_d = 1.185\,nm$, height $0.34 \times 22\,nm$ and surface charge density $-0.02728\mathrm{C\,m}^{-2}$ whose axis aligns with the axis of the simulation box, while the ssDNA molecule was modeled as a cylinder with radius $r_s = 0.838\,nm$, height $0.64 \times 22\,\mathrm{nm}$, and surface charge density $-0.0047\mathrm{C\,m}^{-2}$. These particles were moved along the axis of the nanopore and the net current through the nanopore was measured through

$$I = \int_S F \sum_{i=1}^{2} (z_i N_i) \cdot n dS,$$

where *F* is the Faraday constant, *S* is the cross-sectional area of the nanopore, z_i and N_i are the valence and ionic fluxes of the positive and negative ions respectively, and *n* is the unit normal vector pointed from *S* into the fluid.

Supplementary Figure 12 represents the normalized current drops $\chi = (I - I_0)/I_O$ obtained with the full dielectric membrane ($\varepsilon_\mathrm{m} = 3, \varepsilon_\mathrm{film} = 8$), with an insulating membrane but dielectric coating ($\varepsilon_\mathrm{m} = 0, \varepsilon_\mathrm{film} = 8$), and for an insulating membrane ($\varepsilon_\mathrm{m} = \varepsilon_\mathrm{film} = 0$) with a fixed surface charge density on segments HE and EF estimated from the system with an insulating membrane but dielectric coating, at a voltage of $V_0 = 0.5\,V$. As the current drop is equivalent for the three systems, while the system with an imposed surface charge density on segments HE and EF is less computationally expensive, in the next simulations we will impose these conditions on our membrane.

To allow for a better capture of the negatively charged nucleic acids, the dielectric coating used in our experiments is positively charged. To take this into consideration, we repeat our simulations for both double and single stranded molecules varying the surface charge densities of segments HE and EF. Figure 7a shows the maximum normalized current drop obtained for these molecules at a voltage $V_0 = 0.5\,V$ with different surface charge densities on segments HE and EF. As expected from classical theories where the change in current is a competition between the volume exclusion of ions in the pore by the translocating entity, which decreases the conductivity, and the introduction of new ions brought into the pore by its respective ionic cloud, which increases it, the double stranded molecule presents a bigger current drop than its single stranded counterpart at high ionic strengths ($[\mathrm{KCl}] = 1\,\mathrm{M}$). Increasing the surface charge density of segment EH (inner surface charge density) leads to a higher contribution on surface conductance to the overall ionic current, and therefore both molecules – which translocate far from the surface of the nanopore- present lower current drops at higher inner surface charge distributions. For these situations, the role of the outer surface charge (segment EF) is minimal, as the presence of these molecules happens far enough from the surface to modify either the electroosmotic flow or the ionic concentrations in the corners of the nanopore.

Due to field penetration as well as the high van der Waals interactions of the surface with the exposed bases, a single stranded molecule may pin to the surface, breaking the symmetry of the system. To cater for these events, 3D simulations of the full PNP-NS equations on our nanopore were considered. The built system has already been published in Sensale et al (Supplementary Reference 4). An exhaustive study of the proper meshing was performed to ensure the numerical results are independent of the meshing.

The single stranded molecule is modeled as a half-cylinder with radius r_d, length 0.64×22 nm, and surface charge density $-0.003902\,\mathrm{C\,m^{-2}}$, leading to equal volume and net surface than the model for the single stranded molecule translocating through the middle of the nanopore.

Figure 7b and 7c represents the current drops $\chi = I - I_0$ obtained for the ssDNA translocating through the surface and the dsDNA translocating through the middle, respectively, with inner and outer surface charge densities of $+0.01\,\mathrm{C\,m^{-2}}$. On Supplementary Figure 13, the normalized current drops are presented for different values of the inner and outer surface charge densities. At higher surface charge densities, molecules translocating through the surface present larger current drops, as suggested by Sensale et al (Supplementary Reference 4). While the current drop of molecules translocating through the surface are independent of the outer surface charge, the effect of molecules translocating through the surface is not negligible, as the dynamics of ions near the corner of the nanopore change with changes in the surface charge.

For each simulation, an initial mesh heavily refined on narrow regions, near the translocating agents, and near charged surfaces was considered. This mesh was refined through 5 mesh adaptation steps and convergence of the current was assessed during these adaptations. Supplementary Figure 14 presents the convergence of the baseline for 0.5 V. This same procedure was applied to every simulation performed, showing great convergence. For 2D systems, the maximum number of elements solved for was of the order of 500,000 elements for the baseline and 650,000 for the axisymmetric molecules. For 3D system, this number was of the order of 750,000 elements.

Supplementary References

1. Apel, Pavel Y., et al. Shedding light on the mechanism of asymmetric track etching: an interplay between latent track structure, etchant diffusion and osmotic flow. *Physical Chemistry Chemical Physics* **18,** 25421-25433 (2016)

2. Banerjee, S. et al. Slowing DNA Transport Using Graphene-DNA Interactions. *Adv Funct Mater* **25**, 936–946 (2015).

3. Wanunu, M. et al. Rapid electronic detection of probe-specific microRNAs using thin nanopore sensors. *Nat. Nanotechnol* **5**, 807–814 (2010).

4. Sensale, S., Wang, C. & Chang, H.-C. Resistive Amplitude Fingerprints during Translocation of Linear Molecules Through Charged Solid-State Nanopores. *J. Chem. Phys.* **153**, 085102 (2020).
